# Dehydration Stress Memory: Gene Networks Linked to Physiological Responses During Repeated Stresses of *Zea mays*

**DOI:** 10.3389/fpls.2018.01058

**Published:** 2018-07-24

**Authors:** Laetitia Virlouvet, Thomas J. Avenson, Qian Du, Chi Zhang, Ning Liu, Michael Fromm, Zoya Avramova, Sabrina E. Russo

**Affiliations:** ^1^Department of Agronomy and Horticulture, University of Nebraska, Lincoln, NE, United States; ^2^Institut Jean-Pierre Bourgin, INRA, AgroParisTech, CNRS, Université Paris-Saclay, Versailles, France; ^3^LI-COR Biosciences, Lincoln, NE, United States; ^4^School of Biological Sciences, University of Nebraska, Lincoln, NE, United States; ^5^State Key Laboratory of Plant Genomics, Institute of Microbiology, Chinese Academy of Sciences, Beijing, China

**Keywords:** gene co-expression networks, stress memory, drought, photosynthesis, chlorophyll fluorescence, non-photochemical quenching, stomatal conductance

## Abstract

Stress memory refers to the observation that an initial, sub-lethal stress alters plants’ responses to subsequent stresses. Previous transcriptome analyses of maize seedlings exposed to a repeated dehydration stress has revealed the existence of transcriptional stress memory in *Zea mays*. Whether drought-related physiological responses also display memory and how transcriptional memory translates into physiological memory are fundamental questions that are still unanswered. Using a systems-biology approach we investigate whether/how transcription memory responses established in the genome-wide analysis of *Z. mays* correlate with 14 physiological parameters measured during a repeated exposure of maize seedlings to dehydration stress. Co-expression network analysis revealed ten gene modules correlating strongly with particular physiological processes, and one module displaying strong, yet divergent, correlations with several processes suggesting involvement of these genes in coordinated responses across networks. Two processes key to the drought response, stomatal conductance and non-photochemical quenching, displayed contrasting memory patterns that may reflect trade-offs related to metabolic costs versus benefits of cellular protection. The main contribution of this study is the demonstration of coordinated changes in transcription memory responses at the genome level and integrated physiological responses at the cellular level upon repetitive stress exposures. The results obtained by the network-based systems analysis challenge the commonly held view that short-term physiological responses to stress are primarily mediated biochemically.

## Introduction

Understanding responses of plants to water limitation is an imperative for maintaining productivity in both agricultural and natural settings in the face of climate change (Porter and Semenov, 2005; Zhu et al., 2010; Lobell and Gourdji, 2012; Trenberth et al., 2014). These responses involve a range of physiological and biochemical mechanisms that have cost-benefit trade-offs in a fluctuating environment (Kozlowski and Pallardy, 2002; Chaves et al., 2003). Although the closure of stomatal pores helps avoid water loss (Schulze, 1986), it decreases CO_2_ and increases O_2_ concentrations in the leaf airspace (Flexas et al., 2004, 2007). In addition to direct limitation of CO_2_ availability to chloroplasts, drought stress also causes perturbations in photosynthetic metabolism and biochemistry that reduce carboxylation (Lawlor and Cornic, 2002; Chaves et al., 2003) and cause oxidative damage, which can ultimately lead to cell death (Chaves and Oliveira, 2004; Avenson et al., 2004; Baker et al., 2007; Lawlor and Tezara, 2009).

To prevent damage under hydraulic conditions unfavorable for carbon fixation, electron transfer can be shifted to alternative pathways (Ruuska et al., 2000) and protection involves a composite of processes collectively known as non-photochemical quenching (NPQ) (Müller et al., 2001). The predominant component of NPQ, energy dependent quenching, or *qE* (Crofts and Yerkes, 1994) harmlessly dissipates excessively absorbed energy as heat and is characterized by rapid reversibility driven by pH changes in the thylakoid lumen (Kramer et al., 1999; Müller et al., 2001; Mittler, 2002; Baier and Dietz, 2005). Regulation of *qE* has been shown to involve concentration changes in inorganic phosphate (*P*_i_) (Takizawa et al., 2008) and to depend on ATP synthase in the chloroplast (Kohzuma et al., 2009). The integrated regulation of these processes is poorly understood, but is thought to be coordinated by hormones, such as abscisic acid and other metabolic and signaling pathways (Parent et al., 2009; Tardieu et al., 2010).

Plants often experience repeated sublethal water stresses, with intervening water-recovery periods over the course of a growing season (Slatyer, 1967). Their phenotypically plastic responses to such temporal variation presumably increase long-term survival and productivity despite repeated stress (Nicotra and Davidson, 2010; Cavanagh and Kubien, 2014). Several lines of empirical evidence support this conjecture. Pre-exposure to stressors alters responses to subsequent stressors (Bruce et al., 2007; Byun et al., 2014; Ding et al., 2014; To and Kim, 2014; Fleta-Soriano and Munné-Bosch, 2016).

Horticultural practices, such as drought hardening, enhance dehydration tolerance and photochemical efficiency under future unfavorable conditions by pre-exposing plants to sublethal stress (Turner, 2003; Byun et al., 2014). These observations suggest that plants have some form of “memory” that alters their responses to a subsequent stress (Bruce et al., 2007; Waters et al., 2008). Evidence of such memory has been demonstrated in *Arabidopsis thaliana* and *Zea mays*. Plants subjected to repeated cycles of dehydration stress, alternating with periods of full-watered recovery, exhibited both transcriptional and physiological memory responses, including reduced water loss rate, during a subsequent dehydration stress, compared to plants experiencing dehydration stress for the first time (Ding et al., 2012, 2014). However, it is not known whether other aspects of photosynthetic metabolism and biochemistry that change during dehydration stress also display memory, nor is it known how these responses are regulated by changes in gene expression to adjust the plant phenotype optimally to repeated cycles of stress and recovery.

While stress memory may be protective, there are likely costs associated with altered metabolism and lost opportunities for resource assimilation (Crisp et al., 2016). While such physiological adjustments are ultimately regulated by the expression of genes involved in dehydration stress response (Hayano-Kanashiro et al., 2009; Lu et al., 2011; Krishna-Reddy et al., 2014), we reason that, in a fluctuating environment, stress memory should act to avoid potentially catastrophic losses due to inadequate stress responses, but return to maximal functioning when favorable conditions resume. In repeated dehydration stress/rehydration cycles, two types of gene response groups were revealed in *A. thaliana* and *Zea mays* (Ding et al., 2012, 2013, 2014): genes that produced similar levels of transcripts in response to each stress, after returning to the initial pre-stressed levels during rehydration (non-memory genes) and genes that in a subsequent stress exhibited a transcriptional response significantly different from the response in the first stress (memory genes). It is not known how transcriptional memory is translated into an integrated physiological response to repeated dehydration stress. However, discovering these mechanisms is critical for understanding stress memory and identifying key genes and pathways to increase drought-resistance of agricultural crops.

**FIGURE 1:**
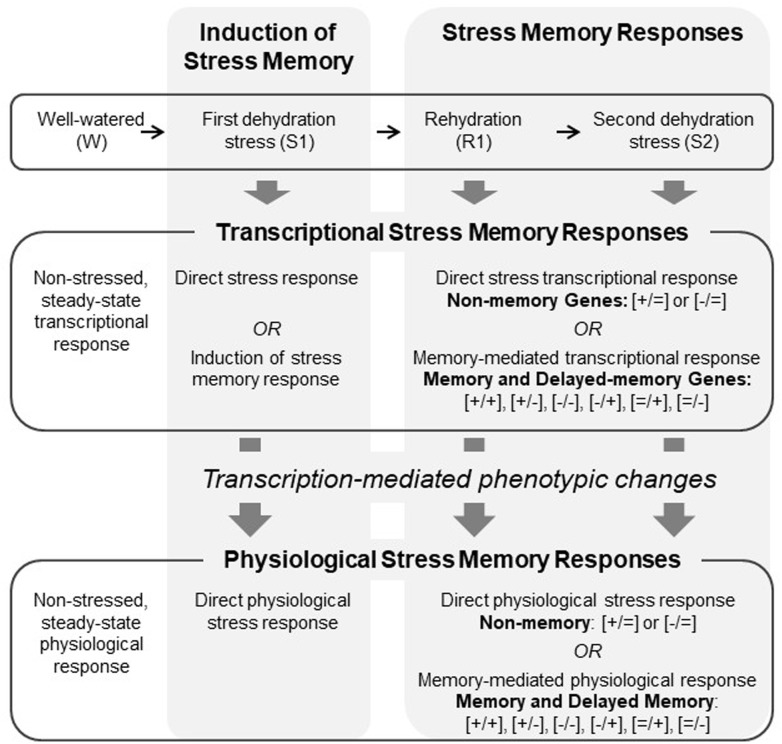
Conceptual framework showing the coordination between transcriptional and physiological responses involved dehydration stress memory.

Our framework (**Figure 1**) posits that the dehydration stress memory response may be thought of as a system of coordinated changes stimulated by an initial dehydration stress that precipitates altered gene expression that underlies, directly or indirectly, physiological responses to achieve a new state of homeostasis due to memory. Many biological processes are controlled by complex gene networks (D’haeseleer et al., 2000; Xiong and Zhu, 2001), and so our framework further posits that the activity of memory genes can be grouped into subsystems, each of which may respond to endogenously and exogenously driven changes in the plant to engender particular physiological responses. We used a systems approach to connect whole-genome transcriptional patterns to leaf physiological responses to repeated exposures to dehydration stress in maize. Specifically, our goals were: (1) to identify sets of genes that exhibit coordinated changes in gene expression under these conditions; (2) to establish whether physiological processes involved in photosynthesis and photoprotection display behavior consistent with memory when exposed to repeated dehydration stress with an intervening rehydration period; (3) to correlate transcriptional and physiological responses in order to identify potential candidates for key genes mediating dehydration stress memory; and (4) to identify putative functions of key genes that may point to particular physiological processes that are most strongly involved in dehydration stress memory. Co-expression network analysis was used to identify modules of co-expressed genes and to determine how they correlated with drought-related physiological parameters. Gene ontology analysis was used to examine whether presumed functions of genes in modules matched expectations based on the network correlations between gene expression and physiological parameters.

## Materials and Methods

Our materials and methods are described in detail in **Appendix S1** and summarized here.

### Gene Transcriptional Analysis

Data from the whole-genome transcriptome analyses of two biological replicates of maize plants exposed to three consecutive dehydration stresses (amounting to six whole-genome RNA seq analyses) were used for the network correlation analyses. These data were previously published in Ding et al. (2014). Genes that in the first stress (S1) and under repeated stresses (S2, S3) were similarly expressed were categorized as non-memory; genes that in S2 or S3 produced transcripts at significantly different levels from the levels produced in S1 were categorized as memory, as described in detail below.

Bioinformatics analysis of transcriptome sequencing data of watered (W), S1, and S2 samples was performed for two biological replicates per sample. The distribution of raw and mapped reads for the two samples is available in Ding et al. (2014, Supplementary Files), and the quality of the replicates (for each of the three samples) was determined as described in detail therein. Genes were classified as being significantly differentially expressed when all three of the following conditions were met: *q* ≤ 0.05; |log_2_(fold change)|≥ 1; and the FPKM-normalized expression value of at least one sample out of the two must be larger than the 25th percentile of all FPKM values within that sample. This additional check for significance (besides *q*-value and fold change) filters out genes that have very low expression in both samples, despite having statistically significant differential expression. For all memory genes, the significant differences in expression were displayed between the S1 and S2 treatments, while the differences between S2 and S3 were not significant. These transcription patterns indicated the memory responses occurred after S1 and were fully displayed in S2. Consequently, the physiological responses measured here were in W, S1, R1, and S2, and transcriptional responses are reported for W, S1, R1, and S3.

### Classification of Stress Memory Response Types

According to our operational definition, a memory response implies that changes in gene expression or in a physiological parameter induced by repeated dehydration stresses (S2 or S3) are significantly different from responses displayed in the first stress (S1). Whole transcriptome data were from W, S1, and S3, whereas physiological data were collected at W, S1, R1, and S2. Transcript analysis of selected genes showed similar expression responses in S2 and S3, and so we considered these stages to be comparable for the transcript and physiological data. Among the 3740 memory genes in *Z. mays*, a subgroup of 2924 genes did not exhibit altered expression in S1 compared to the initial, non-stressed expression in W, but significantly changed (increased or decreased) expression in subsequent stresses. These genes were referred to as delayed memory response genes (Ding et al., 2014) and annotated as [=/−] or [=/+], where the [=] sign indicates the values in S1 were not statistically different from values in W; the second sign indicates the values in S2 were significantly different (lower [−] or higher [+]) than in S1. The other memory response-types are annotated as [+/+], [−/−], [+/−], and [−/+], where the first sign indicates significant difference in S1 *versus* W, and the second sign indicates significant difference in S2 *versus* S1. Non-memory responses are annotated as [+/=] or [−/=] indicating significant positive or negative response in S1 versus W but similar responses in S1 and S2.

### Co-expression Network and Gene Ontology Analyses

Co-expression network analysis was used to identify genes with coordinated transcriptional responses (modules). Among the total of 4986 *Z. mays* genes exhibiting memory and non-memory responses to repeated dehydration stress (Ding et al., 2014), 4710 genes displaying sufficiently high variation (range of FPKM-normalized read counts across a total of six samples for W, S1, and S2 with two biological replicates in each of the three treatments for a given gene >0.05) were identified, and their values were used to construct a correlation matrix using the R package, WGCNA (Langfelder and Horvath, 2008). Dynamic tree cutting was adopted to identify modules with minModuleSize of 30 (Langfelder and Horvath, 2007). Eigengenes were used to cluster all identified modules using Average Hierarchical Clustering analyses (Langfelder and Horvath, 2007). Pairwise distances between modules were calculated from the correlation between eigengenes for each module as an estimate of similarity. A distance of 0.2 was used as a cut-off to group modules into meta-modules.

The correlation coefficients used to construct the co-expression network could be noisy due to our sample size of six for the transcriptomic data, although it has been demonstrated that overall the quality of the transcriptomic data set was good enough to generate reliable results, as shown in Ding et al. (2014). To increase the signal-to-noise ratio and reduce the false positive rate, during network construction, we used a high threshold to fill out genes with low expression levels or high fluctuations, and only genes with high correlation were considered to belong to the same module. This high threshold for data analysis reduced the false positives significantly and facilitates obtaining robust results.

Gene ontology (GO) (Ashburner et al., 2000; Bard and Rhee, 2004) and term enrichment analyses using AgriGO (Du et al., 2010) were performed to identify putative functions of dehydration stress memory genes in the network analysis. Genes sharing the same GO term are considered to be involved in similar biological processes, allowing us to identify the putative functions represented in each module. Statistical analysis was performed using Fisher’s exact test with subsequent adjustment for multiple testing by calculating the false discovery rate (FDR) as implemented in AgriGO. The minimum number of mapping entries was set to 5, and the list of genes from the maize genome AGPv3 release 5a database was used as a reference^1^.

### Plant Growth and Treatments

All measurements were performed on 2-week old greenhouse-grown *Z. mays* L. (cultivar B73) seedlings. Handling of plants after removal from soil and dehydration stress-rehydration treatments were performed as previously described (Ding et al., 2014). Briefly, whole seedlings were air-dried for 90 min (first dehydration stress, S1) followed by 22 h of full rehydration with roots in water in covered trays (R1). R1 plants were again exposed to air-drying for 90 min (S2), followed by another rehydration treatment (R2). In order to avoid confounding wounding responses from cut tissue, different sets of plants were used for gas exchange and chlorophyll fluorescence, versus for the transcriptome, RWC, abscisic acid, and chlorophyll measurements, since the latter require leaf harvesting.

### Physiological Parameters

In each dehydration treatment stage, we measured several physiological parameters related to water stress (**Table 1**): photosynthetic gas exchange, pulse-amplitude-modulation (PAM) chlorophyll *a* fluorescence (Schreiber et al., 1995), foliar relative water content (RWC), and foliar abscisic acid (ABA) and chlorophyll concentrations. The rates of net photosynthesis (*P*_N_, μmol CO_2_ m^−2^ s^−1^), stomatal conductance (*g*_s_, mmol H_2_O m^−2^ s^−1^), transpiration (*E*, mmol H_2_O m^−2^ s^−1^), and PAM chlorophyll *a* fluorescence were measured simultaneously on the fourth leaf of seedlings at W, S1, R1, and S2 stages using a LI-6400XT infrared gas analyzer with a 6400-40 fluorescence chamber, with a uniform integrated LED light source and PAM fluorometer (LI-COR Inc., Lincoln, NE, United States). These parameters were simultaneously recorded, and a mean for each parameter for each of ten plants at each stage was estimated from 13 measurements at steady state, taken every 25 s over 5 min. In estimating chlorophyll fluorescence parameters (Baker et al., 2007), we used approaches described in the literature (Edwards and Baker, 1993; Lal and Edwards, 1996; Maxwell and Johnson, 2000; Kramer et al., 2004a). We assumed for *φPSII* (photosystem (PS) II operating efficiency) that the proportion of Q_A_ (reaction centers in the oxidized state) was between 0 and 1 (Genty et al., 1990) and, for *F*_v_′*/F*_m_′ (PSII maximum efficiency), that the proportion of *Q*_A_ was 1. We estimated two expressions of energy-dependent quenching based on different assumptions of PSII oxidation state, *qE* and *φqE* (Ahn et al., 2009), as well as the ratio, *qE*/*ETR*, a proxy expression describing a phenomenon termed *qE* sensitivity (Kramer et al., 2004b). We also calculated the composite parameters, *φPSII/φCO_2_* and *ETR*/*A*_G_, which have been associated with drought responses (Edwards and Baker, 1993).

**Table 1:** Physiological parameters and their units estimated for *Zea mays* leaves during dehydration stress treatments.

Parameters	Name	Units
RWC	Relative water content	%
*g*_s_	Stomatal conductance	mmol H_2_O m^−2^ s^−1^
*E*	Transpiration	mmol H_2_O m^−2^ s^−1^
*C*_i_	Leaf internal CO_2_ concentration	μmol CO_2_ mol^−1^
*P*_N_	Photosynthetic rate	μmol CO_2_ m^−2^ s^−1^
*A*_G_	Gross Photosynthetic rate	μmol CO_2_ m^−2^ s^−1^
*ETR*	Electron transport rate in PSII	
*φPSII*	Quantum yield of photochemical energy conversion	
*F*_v_′*/F*_m_′	Efficiency of the PSII open reaction centers	
*qP*	Coefficient of photochemical quenching	
*φCO_2_*	Quantum yield of CO_2_ assimilation	
*qE*	Coefficient of energy-dependent quenching	
*φqE*	Quantum yield of energy-dependent non-photochemical quenching	
ABA	Foliar abscisic acid concentration	pmol g^−1^ DW
Chlorophyll	Foliar chlorophyll concentration	mg g^−1^ DW

The foliar abscisic acid (ABA) assay was performed as in Liu et al. (2014). Four technical readings in each treatment were used to obtain a mean, which were averaged across four plants within the W, S1, R1, and S2 stages. Foliar chlorophyll content was measured at the W, S1, R1, and S2 stages. Leaf disks were immediately ground in liquid nitrogen, homogenized in 80% (v/v) acetone, and absorbance (*A*) was read at 663 and 645 nm. The total chlorophyll content (mg g^−1^ DW) was calculated as [(8.02 × *A*663 nm) + (20.2 × *A*645 nm)]/*DW*, where *DW* is the dry weight of ground leaf material (Arnon, 1949). Two different sets of plants were analyzed on two dates, each of which had three replicate pools per treatment, with each pool containing the same tissue amount from five plants from a treatment.

### Analysis of Physiological Parameters and Correlation Between Transcriptional and Physiological Dehydration Stress Responses

To quantify changes in physiological parameters across dehydration treatments, we used Student’s *t*-tests. Chlorophyll concentrations were analyzed using a linear mixed-effect model, with experiment date as a random intercept, and differences due to treatment analyzed using type III tests of fixed effects with degrees of freedom determined by the Satterthwaite method as implemented in R statistical software (R Core Development Team, 2011) using the packages *lme4* (Bates et al., 2014) and *lmerTest* (Kuznetsova et al., 2014).

The novel component of our study is that the previously published transcriptomic data (Ding et al., 2014) were integrated with the detailed plant physiological data using bioinformatic and gene expression network analyses in order to investigate how changes in gene expression correlated with changes in specific physiological processes during response to repeated drought stress. To do so, the eigengene of each module from the co-expression network analysis was correlated with the response levels across dehydration treatments for each physiological parameter as a measure of the level of coordination between genes in that module and physiological responses.

## Results

### Gene Co-expression Networks

**FIGURE 2:**
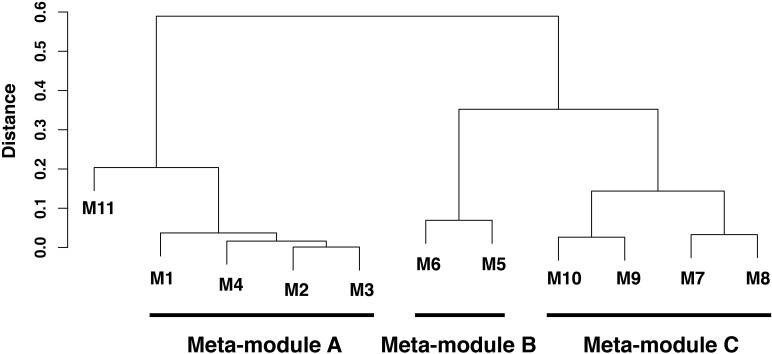
Hierarchical clustering analysis of co-expressed genes in leaves of *Zea mays* seedlings in response to repeated dehydration stress and rehydration treatments. Three meta-modules (modules M1–M10) and one single module (module M11) were identified based on the dendrogram of 11 discovered modules identified by a hierarchical clustering analysis based on average linkage method for eigenvectors of these modules (eigengene). The height of branches is the distance between two corresponding eigenvectors, which is one minus their correlation coefficients. An eigenvector is the first principal component of the corresponding module, and the eigenvector characterizes the gene expression pattern for all genes in this given module. Two modules with lower distance in the dendrogram have higher similarity of gene expression patterns.

**FIGURE 3:**
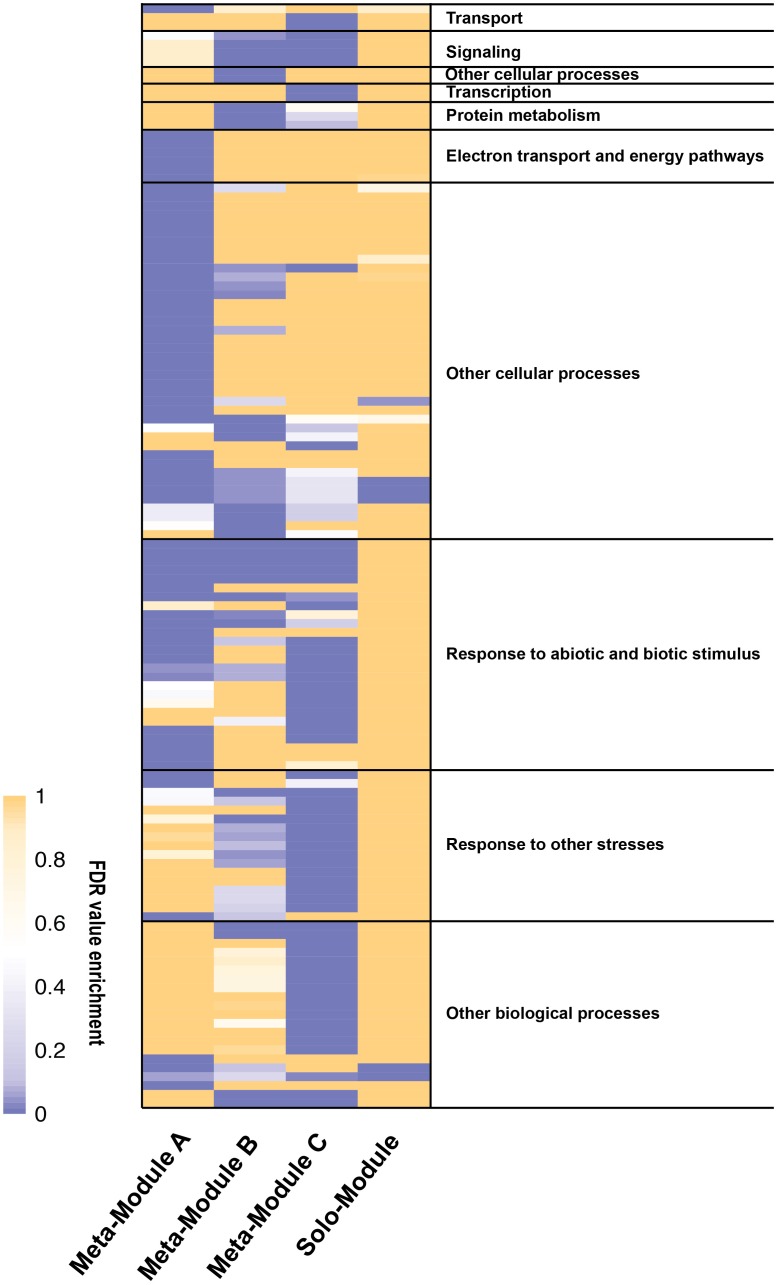
Heatmap of gene ontology (GO) enrichment analysis of the meta-modules genes expressed in *Z. mays* under repeated dehydration stress and rehydration treatments. The GO terms enriched significantly (FDR < 0.01) for at least one meta-module have been grouped accordingly to the GO slim list established for *Arabidopsis thaliana*. See **Supplementary Table S3** for the entire list of the GO terms. The legend shows the color scaling with FDR values.

Among the 4986 maize genes responding to repeated dehydration stress (Ding et al., 2014), co-expression network analysis identified 11 gene modules; 10 of them were grouped into three clusters (meta-modules), each containing four, two, and four modules, plus one module (M11) that did not group with any other module and remained as a ‘solo module’ (**Figure 2** and **Supplementary Table S2**). The co-expressed stress-responding genes in these differed broadly in predominant GO slim classification functions revealed in GO term analysis (**Figure 3** and **Supplementary Table S3**).

### Meta-Module A Is Enriched in Genes Involved in Photosynthesis and Fluorescence Mechanisms

Meta-module A, represented by 2757 co-expressed genes, comprised four highly correlated modules, M1 (1588 genes), M4 (925 genes), M3 (205 genes), and M2 (39 genes) (**Supplementary Table S2**). Of these, 95% (2613 genes) exhibited a delayed memory response, including genes up- (1466 [=/+]) and genes down- (1147 [=/−]) regulated in S3. The remaining 5% (143 genes) showed other memory responses; none were non-memory genes. Meta-module A was enriched in genes encoding proteins implicated in electron transport and energy pathways, in pigment, carbohydrate and glycoside metabolic processes, and in response to biotic and abiotic stimuli including light (**Figure 3** and **Supplementary Table S3**). While no enrichment in particular functions was identified for the combined 244 genes of modules M3 and M2 (**Supplementary Table S3**), genes implicated in the CBB cycle, in light harvesting, electron transport, non-photochemical quenching, and in overall photosynthesis, were over-represented in modules M1 and M4 (**Supplementary Table S3**). The expression patterns of 94% of the M1 and M4 genes corresponded to delayed memory responses (**Supplementary Table S2**). Effectively, in module M1, 47 [=/−] delayed memory response genes were implicated in light harvesting, including proteins for PSI and PSII, chlorophyll A/B binding proteins, electron transport and cyclic electron flow proteins. In addition, 13 [=/−] delayed memory response genes encoding enzymes for the CBB cycle, including ribose-5-phosphate isomerase, glyceraldehyde-3-phosphate dehydrogenases A and B, phosphoglycerate kinases, phosphoribulokinase, and ribulose bisphosphate carboxylases were also clustered in M1 (**Supplementary Table S2**).

### Meta-Module B Is Enriched With House-Keeping Genes

Meta-module B containing modules M5 and M6 was defined by 357 (79%) co-expressed memory and 95 (21%) non-memory genes (**Figure 2** and **Supplementary Table S2**). The meta-module was largely enriched in house-keeping genes and genes involved in signaling, cell wall organization, protein modification, and response to stimuli (**Figure 3** and **Supplementary Table S3**).

### Meta-Module C Is Linked to Regulation of Diverse Processes

Meta-module C was defined by 1125 co-expressed genes clustered in modules M7, M8, M9, and M10 (**Figure 2** and **Supplementary Table S2**). Module M7, containing 602 (93%) and 44 (7%) co-expressed non-memory and memory genes. In general, meta-module C was enriched by genes implicated in transport, signaling, transcription, regulation of numerous biological processes and response to stimuli (**Figure 3** and **Supplementary Table S3**).

### The Solo Module Is Enriched in Small Molecule Biosynthetic Process

The solo module (M11) grouped 241 co-expressed genes: 128 (52%) genes were categorized as memory, 36 (15%) genes as delayed memory, and 77 (32%) genes as non-memory response genes (**Supplementary Table S2**). The GO analysis indicated enrichment in small molecule biosynthetic process representing proteins involved in lipid metabolism, stress responses, and in producing intermediates of the C4 metabolic pathway, including allosteric inhibitors (Gonzalez et al., 1986) (**Figure 3** and **Supplementary Table S3**).

### Changes in Physiological Parameters in Response to Repeated Dehydration Stress

**FIGURE 4:**
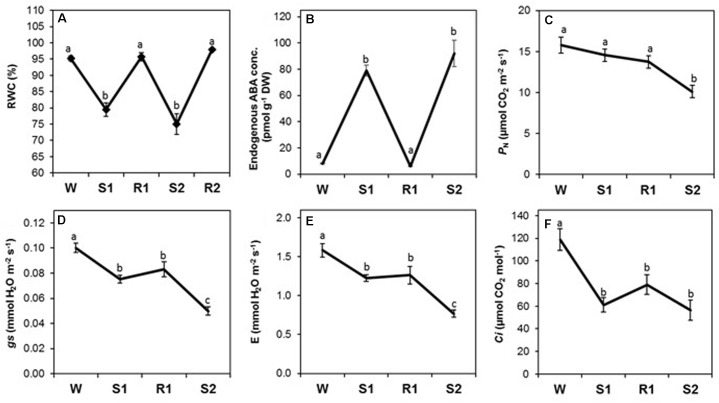
Changes in foliar relative water content (RWC), abscisic acid (ABA) concentration, and gas exchange parameters during successive dehydration stress and rehydration treatments in *Z. mays*. **(A)** RWC, **(B)** ABA concentration, **(C)** net photosynthesis rate (*P*_N_), **(D)** stomatal conductance (*g*_s_), transpiration rate **(E)**, and **(F)** internal CO_2_ concentration (*C*_i_*)* were measured under the well-watered condition (W), after the first (S1) and second (S2) dehydration stresses, and after rehydration (R1; R2 only for RWC). Values are means ± SE (*n* = 10, 10 and 4 plants per treatment for RWC, gas exchange parameters, ABA concentration, respectively). Different letters correspond to statistically significant differences between treatments (*P* < 0.05).

Foliar RWC decreased significantly in S1 and S2, but returned to pre-stressed watered (W) levels after each rehydration (R1 and R2, **Figure 4A**), consistent with a non-memory ([−/=]) response. Foliar ABA also exhibited a non-memory response, but its [+/=] pattern was opposite to that of RWC (**Figure 4B**), as expected, since increases in ABA trigger closure of stomatal pores during dehydration stress (Lim et al., 2015).

The rates of stomatal conductance (*g*s) and transpiration (*E*) declined from W to S1, as expected, but did not return to levels observed in W after rehydration in R1, despite full restoration of RWC and a significant decline in ABA during R1 (**Figures 4A,B**). Both declined significantly to levels lower in S2 compared to S1 (**Figures 4D,E**), exhibiting a stress memory response [−/−]. The net rate of photosynthetic CO_2_ fixation (*P*_N_) did not initially respond strongly to dehydration stress, and it was only by S2 that *P*_N_ had declined to rates significantly lower than in W (**Figure 4C**), consistent with a delayed [=/−] memory response. The CO_2_ concentration in the leaf internal airspace (*C*_i_) declined precipitously from W to S1, as expected due to closure of stomatal pores (**Figure 4F**). During R1 and S2, *C*_i_ remained at similar levels, displaying a non-memory [−/=] response, likely a result of the combined effect of reduced *g*s being buffered by reduced *P*_N_ in S2.

**FIGURE 5:**
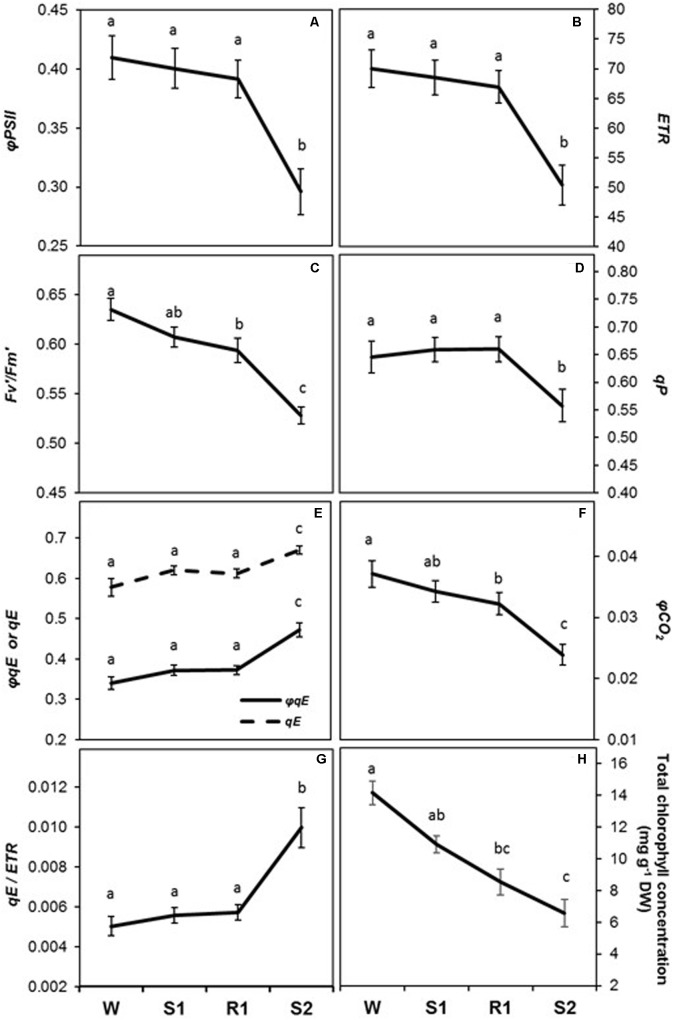
Changes in photosynthetic biochemistry and foliar chlorophyll concentration during successive dehydration stress and rehydration treatments in *Z. mays*. **(A)** *φPSII*, **(B)** electron transport rate (*ETR*), **(C)** *F*_v_′*/F_m_*′, **(D)** *qP*, **(E)** *qE* and *φqE*, **(F)** *φCO_2_*, **(G)** *qE*/*ETR*, and **(H)** foliar chlorophyll concentration were measured under the well-watered condition (W), after the first (S1) and second (S2) dehydration stresses, and after rehydration (R1). Fluorescence parameters **(A–G)** were derived from the chlorophyll fluorescence measurements performed simultaneously with gas exchange measurements. See **Table 1** for abbreviations, definitions, and units of fluorescence parameters. Values are means ± SE (*n* = 10 and 6–7 plants per treatment for the chlorophyll fluorescence and concentration, respectively). Different letters correspond to statistically significant differences between treatments (*P* < 0.05).

**FIGURE 6:**
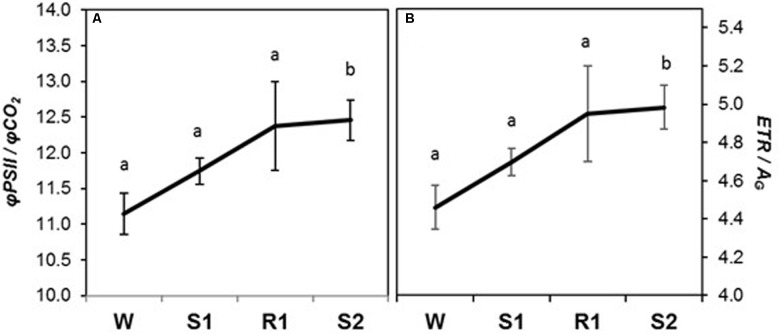
Changes in chlorophyll fluorescence parameters during dehydration stress and rehydration treatments in *Z. mays*. **(A)** *φPSII*/*φCO_2_*, and **(B)** electron transport rate (*ETR*)/gross photosynthetic rate (*A*_G_) were measured under the well-watered condition (W), after the first (S1) and second (S2) dehydration stresses, and after rehydration (R1). Fluorescence parameters were derived from chlorophyll fluorescence measurements performed simultaneously with gas exchange measurements. See **Table 1** for abbreviations, definitions, and units of fluorescence parameters. Values are means ± SE (*n* = 10 plants per treatment). Different letters correspond to statistically significant differences between treatments (*P* < 0.05).

All chlorophyll fluorescence parameters displayed delayed memory responses, as did the ratio of *qE*/*ETR*, a proxy expression for *qE* sensitivity (**Figures 5A–G**). These parameters exhibited their most dramatic changes from R1 to S2. The responses of *φPSII*, *ETR*, and *qP* were similar to that for *P_N_*, in that they declined slightly from W to S1 and only became significantly different from levels at W by S2 ([=/−]) (**Figures 5A,B,D**), whereas *F*_v_′*/F*_m_′ and *φCO_2_* displayed a more noticeable, but still gradual, decline from W to S1 (**Figures 5C,F**). The decline in *F*_v_′*/F*_m_′ and in *qP* between R1 and S2 indicates enhanced NPQ that can account for the observed sharp decline in *φPSII*. Consistent with this, parameters reflecting increases in the amount of energy dissipated via non-photochemical sinks, *qE*, *φqE*, and *qE*/*ETR* (**Figures 5E,G**) remained at similar levels from W to S1, but increased dramatically from S1 to S2 ([=/+]), whereas *qP* declined. Foliar chlorophyll declined continuously from W to S2 (**Figure 5H**), displaying what may best be classified as a memory response ([−/−]). The composite parameters, *φPSII/φCO_2_* and *ETR*/*A*_G_ also showed a delayed memory response ([=/+]) in that only levels at W and S2 were significantly different from each other, although they exhibited a slight increase from W to S1 that was not statistically significant (**Figure 6**).

### Changes in Gene Expression in Response to Repeated Dehydration Stress

Among the genes responding to repeated dehydration stress, a total of 164 genes encoding known dehydration stress-associated proteins were classified into four broad functional categories related to photosynthesis, pigments, stomatal regulation, and production of ABA. Most of them, 119 genes, belonged to the delayed memory category, 19 genes exhibited a memory response, and 26 were non-memory genes (**Table 2**).

**Table 2:** Dehydration stress response genes classified into four broad functional categories related to photosynthesis, pigments, stomatal regulation, and production of ABA.

Category	No. genes	Transcriptional Memory pattern							
		[+/+]	[+/−]	[+/=]	[=/+]	[−/−]	[−/+]	[−/=]	[=/−]
Photosynthesis	105	1	3	2	11	3	0	2	83
Calvin–Benson–Basham Cycle	16	–	–	–	2	–	–	–	14
NADP-ME type	2	–	–	–	2	–	–	–	–
NAD-ME type	2	–	1	–	–	–	–	–	1
PEPC	2	1	–	–	–	–	–	–	1
PEPCK enzyme type	2	–	–	–	1	–	–	–	1
PEPC kinase	4	–	1	–	3	–	–	–	–
Other photosynthesis proteins	4	–	–	1	2	–	–	–	1
Photosystem I	11	–	–	–	–	–	–	–	11
Photosystem II	13	–	–	–	–	2	–	1	10
Cytochrom b6f	4	–	–	–	–	–	–	–	4
Quinone/ferredoxin	5	–	1	1	–	–	–	1	2
Chlorophyll a/b binding protein	16	–	–	–	–	–	–	–	16
Other light photosynthesis proteins	5	–	–	–	–	1	–	–	4
Electron transport	8	–	–	–	–	–	–	–	8
Nonphotochemical regulation	1	–	–	–	–	–	–	–	1
Photosynthesis regulation	10	–	–	–	1	–	–	–	9
**Pigment**	**13**	**0**	**2**	**3**	**3**	**0**	**0**	**1**	**4**
Chlorophyll biosynthesis	2	–	–	–	–	–	–	–	2
Chlorophyll degradation	4	–	1	–	2	–	–	1	–
Carotene	1	–	–	–	–	–	–	–	1
Zeaxanthin biosynthesis	4	–	1	3	–	–	–	–	–
Zeaxanthin/violaxanthin interconversion	2	–	–	–	1	–	–	–	1
**Stomatal regulation**	**35**	**2**	**2**	**12**	**12**	**0**	**1**	**1**	**5**
Channel	6	–	1	3	–	–	–	–	2
Stomatal regulation	1	–	–	–	–	–	–	–	1
Stomatal movement	**28**	2	1	9	12	–	1	1	2
**ABA**	**11**	**1**	**4**	**5**	**0**	**0**	**0**	**0**	**1**
ABA biosynthesis	6	–	3	3	–	–	–	–	–
ABA degradation	5	1	1	2	–	–	–	–	1
**Total genes**	**164**	**4**	**11**	**22**	**26**	**3**	**1**	**4**	**93**

Ninety four genes (79%) of the delayed memory response group encoded proteins associated with the light-dependent and carbon fixation reactions of photosynthesis (**Table 2**), including proteins implicated in electron transport in PSI and PSII (e.g., cytochrome *b_6_f* and quinone/ferredoxin), chlorophyll a/b binding, signaling and redox regulation, as well as proteins involved in the Calvin–Benson–Bassham (CBB) cycle (**Table 2** and **Supplementary Table S1**). Delayed memory responses were displayed also by seven of the 13 genes associated with the metabolism of pigments and NPQ functions. In particular, two chlorophyll catabolic genes activated in S3 (delayed memory [=/+] genes), and two chlorophyll biosynthesis genes downregulated in S3 ([=/−] delayed memory genes) displayed patterns consistent with the decline in chlorophyll levels observed during the dehydration stresses (**Figure 5G**). Two genes implicated in the xanthophyll cycle, violaxanthin de-epoxidase (VDE, npq1) and zeaxanthin epoxidase (ZE, npq2) also displayed delayed memory, complementing each other’s memory responses ([=/−] and [=/+], respectively; **Supplementary Table S1**). These changes in transcription suggest violaxanthin levels would increase, whereas zeaxanthin would decrease in subsequent stresses, creating an apparent paradox, as conversion of violaxanthin to zeaxanthin is involved in energy-dependent NPQ, which increased during dehydration stress. However, the gene coding for the γ subunit of CF_0_CF_1_ ATP synthase, displayed a [=/−] memory response. Its reduced expression in S3, implying putative reductions in amounts of ATP synthase, would ultimately increase *qE* (Kohzuma et al., 2009) (**Supplementary Table S2**).

Seventeen genes (49%) of the 35 dehydration stress-responding genes implicated in the biogenesis, distribution, and movement of guard cells, including ion channel proteins that drive changes in stomatal apertures, belonged to the delayed memory category (**Table 2**). Of the remaining 18 genes, 13 exhibited non-memory and five exhibited other memory-type responses in S3 (**Table 2**). Of note is that members of the same gene families involved in ABA-dependent changes in stomatal aperture, displayed a broad spectrum of transcriptional responses, despite encoding proteins with similar structure and predicted biochemical functions (**Supplementary Table S1**). The increased transcription in S3 of the genes implicated in calcium-mediated signaling of ABA-dependent pathways and in ABA-signaled stomata closure (Mori et al., 2006; Vahisalu et al., 2008; Dreyer et al., 2012; Imes et al., 2013; Yu et al., 2014), suggested they would stimulate stomatal closing. In contrast, the expression of genes driving stomatal opening [i.e., inward potassium channel protein, KZM1 (Guo et al., 2002) and two nitrate transporters (Philippar et al., 2003) were significantly downregulated in S3].

Ten ABA metabolic genes were induced in S1; upon repeated stress, five displayed non-memory, and five displayed memory responses (**Table 2**). Among the latter, two genes encoding ABA-anabolic and two genes encoding ABA-catabolic enzymes declined in expression (displaying a [+/−] response pattern), while one (for a catabolic abscisic acid 8′-hydrolase) was super-induced ([+/+]) (**Table 2** and **Supplementary Table S1**). Since both ABA-synthesizing and ABA-degrading biochemical pathways were activated when plants experienced dehydration stress, the results suggest that the foliar ABA levels were determined by the combined effect of both ABA anabolism and catabolism. One ABA degrading gene displayed a [=/−] delayed memory response.

### Correlation Between Transcriptional and Physiological Responses

**FIGURE 7:**
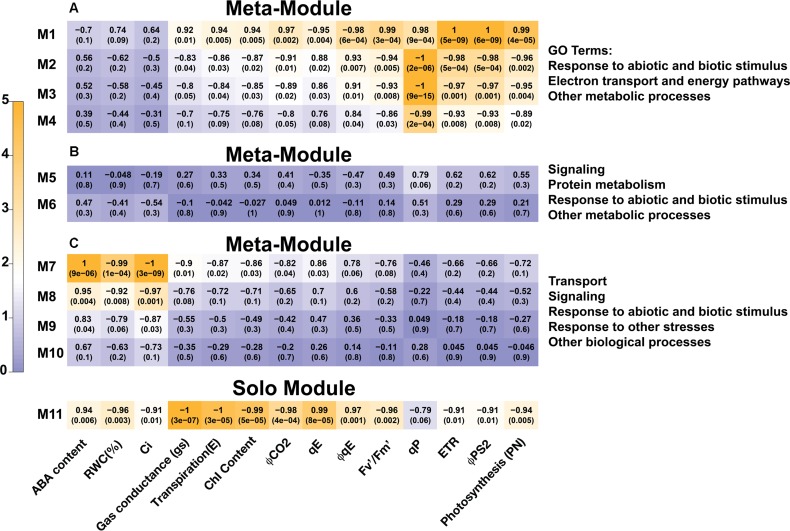
Correlations among gene co-expression modules and physiological parameters in *Z. mays* exposed to repeated dehydration stress and rehydration treatments. Each row corresponds to a module, and each column to a physiology parameter. One cell indicates the correlation between the eigenvector of this given module and the corresponding physiology parameter. In one cell, the corresponding *p*-value is shown below the correlation coefficient. The colors of cells, changing gradually from blue to orange to red, indicate the significance of correlations among module eigenvectors and physiological parameters, with the legend showing the correspondence of colors to −log_10_(*p*-values).

The strength of correlation between physiological parameters and the expression of genes in modules exhibited wide variation (**Figure 7**). Strong correlation of specific modules with particular physiological responses suggests that physiological changes involved in dehydration stress memory are associated with distinct networks of genes operating in a coordinated way.

Expression of genes in meta-module A correlated strongly with photosynthesis and chlorophyll fluorescence parameters (**Figure 7**). The constituent modules, however, differed in the strength of correlations with individual physiological parameters: module M1 correlated strongly with φ*PSII*, *ETR, P*_N_, φ*qE*, and *F*_v_′*/F*_m_′, and less strongly with φ*CO_2_, qE*, and *qP*; all correlations, except with *qE* and φ*qE*, were positive. The M4, M3, and M2 modules also correlated with φ*PSII*, *ETR*, and *P*_N_, as well as *qP*, but, in contrast to M1, the correlations were negative and weaker. However, no strong correlations of M5 and M6 modules of meta-module B with the physiological parameters measured here were found (**Figure 7**). Interestingly, only module M7 from meta-module C correlated negatively with *C*_i_ and *RWC* and positively with ABA (**Figure 7**). Weaker correlations with *C*_i_ and ABA were displayed by the M8 module, while no significant correlations with the responses studied here were found for modules M9 and M10. The solo module, M11, displayed negative correlations with stomatal conductance (*g*_s_), transpiration (*E*), φ*CO_2_*, and chlorophyll (*Chl*) content, a positive correlation with *qE*, and a weaker positive correlation with φ*qE* (**Figure 7**). Thirty-one [−/−] and 97 [+/+] memory genes in the solo module correlated positively or negatively, respectively, with the [−/−] memory responses displayed by the *g*_s_ and *E* parameters (**Figures 4D,E**).

## Discussion

Being sessile, plants cannot escape environmental variability, and so have evolved integrated, plastic responses to maintain fitness despite fluctuating biotic and abiotic stresses. Although these mechanisms are becoming better understood, much remains to be discovered regarding the role of stress memory. Genome-wide screening of dehydration stress-responding genes in combination with detailed physiological measurements revealed that widespread transcriptional changes are an important component of physiological dehydration stress memory. Our discovery challenges the commonly held view that short-term physiological responses to stress are primarily mediated biochemically. Moreover, we discovered that processes of known importance in plant drought response displayed contrasting memory patterns: stomatal conductance declined with initial dehydration and was further reduced in the subsequent stress, whereas photosynthesis and NPQ showed delayed memory with significant changes only in the subsequent stress. Adjustments in translation may also occur, but, to our knowledge, ours is the first study to demonstrate that the sophisticated physiological memory responses to repeated dehydration stress are, apparently, driven by widespread transcriptional changes in genes implicated in diverse photosynthetic and photoprotective functions. While they clearly interact, the relative importance of expression-independent versus dependent mechanisms in dehydration stress memory is unknown, but likely depends on the timescale of fluctuations in water stress.

Dehydration-induced transcriptional changes were clearly organized in distinct modules of co-expressed genes and were correlated to varying degrees with photosynthetic and photoprotective responses to repeated stresses. It is important to note that the expression of genes that did not correlate with any physiological parameter measured here had mainly generalized, house-keeping-type functions. Most genes, however, showed strong, consistent correlations with particular physiological parameters, implying that some physiological responses involved in dehydration stress memory were associated with expression changes in distinct networks of interdependently operating genes. In contrast, the expression patterns of genes that displayed strong, but divergent, correlations with several physiological parameters suggested that they might be less integral to particular responses or might integrate signals across groups of co-responding genes. Although many of these genes have not been functionally annotated, their high transcriptional connectivity and correlations with particular physiological parameters allow us to map how they interact to mediate the complex phenotype of dehydration stress memory.

### Memory Responses Limiting Gas Exchange and Protecting the Photosynthetic Apparatus

Previous research examining dehydration stress memory revealed that the fresh weight of maize seedlings declined more slowly in subsequent, relative to the first, dehydration stresses (Ding et al., 2014). Water loss through transpiration is governed by ABA-mediated changes in stomatal apertures (Schulze, 1986; Lim et al., 2015). In *Aptenia cordifolia*, a CAM plant, ABA showed differences in double-stressed plants compared with plants challenged with drought for the first time (Fleta-Soriano et al., 2015). Despite the presence of multiple copies of ABA-metabolic genes in the maize genome, only half of them participated in dehydration stress responses, and the observed transcription patterns suggested a complex interaction among ABA-metabolizing activities. The lack of super-induced ABA levels in successive stress cycles rules out a model wherein retention or increase of ABA was responsible for the super-induced transcript levels of other memory genes or for the memory responses of *g*_s_ and *E*. The memory response of conductance is likely due to a mechanism maintaining reduced stomatal apertures even after recovery, as also found for *Arabidopsis* in an analogous memory response (Virlouvet and Fromm, 2015). Moreover, the recovery of conductance may decrease or require longer times as the severity of the dehydration stress increases (Miyashita et al., 2005; Efeoğlu et al., 2009).

Drought stress is typically described as resulting in diminished supplies of CO_2_ to chloroplasts due to reduced stomatal conductance, thereby causing diminished carboxylative demand and energy production, as evidenced by concurrent decreases in CO_2_ assimilation and *ETR* (Lal and Edwards, 1996; Medrano et al., 2002). Such changes could result from purely biochemical mechanisms; however, the transcript and GO term analyses showed dramatic evidence for delayed memory responses of many genes involved in photosynthesis. The reduced stomatal conductance between R1 and S2, apparently, did not cause a decrease in either carboxylative or energy production capacities *per se*, that is, by reductions in chloroplastic levels of CO_2_, since *C*_i_ levels remained constant between R1 and S2. Instead, transcriptional changes, including diminished expression of CBB and electron transfer-associated proteins, were elicited that were associated with a coordinated decrease in carboxylative and energy producing capacities, as shown by as shown by the reduction in both *P*_N_ and *ETR*. The non-memory response of *C*_i_ is thus likely due to compensatory changes in stomatal conductance and the coupled carboxylation and *ETR* reactions that coordinate CO_2_ assimilation. The diminished carboxylative and energy-producing capacities were accompanied by transcriptional-level decreases in energy-capture capacity, as suggested by down-regulation of light harvesting (i.e., chlorophyll-binding) proteins and chlorophyll biosynthesis enzymology, ultimately reducing foliar chlorophyll.

A novel aspect of this diminished energy capture-capacity is the first evidence supporting the hypothesis that the multifaceted photoprotective responses involved in dehydration stress memory are explicitly controlled at the transcriptional level. To maximize CO_2_ assimilation while minimizing photodamage in a fluctuating environment, plants must flexibly adjust *qE*, the predominant component of NPQ (Avenson et al., 2005), and this ability has recently been shown to increase crop productivity (Kromdijk et al., 2016). In studies of drought (Avenson et al., 2005; Kohzuma et al., 2009; Kanazawa et al., 2017), biochemical perturbation of the carboxylative reactions was accompanied by diminished *ETR*, and yet *qE* remained as, if not more, robust in comparison to control conditions. In effect, *qE* became more sensitive to *ETR* under biochemical perturbation of the carboxylative reactions. In a biochemical model for the mechanisms underlying drought-induced increases in *qE* sensitivity, chloroplastic ATP synthase emerged as a central player (Kramer et al., 2004a). This model, however, did not invoke any transcription-mediated phenomena. It proposed that the relative resistance of chloroplastic ATP synthase to proton efflux increases via kinetic constraints imposed by inorganic phosphate (*P*_i_) limitation (Takizawa et al., 2008) and/or diminished levels of ATP synthase (Kohzuma et al., 2009). The resulting reduced proton efflux lowers the pH of the thylakoid lumen causing: (1) the rate of electron transfer through the cytochrome b6f complex, and consequently *ETR*, to slow down so as to match the diminished carboxylative capacity and (2) the pH-dependent mechanisms controlling the magnitude of *qE* (i.e., protonation of Psbs and activation of VDE) to be enhanced. The net result is that lower fluxes of *ETR* are capable of maintaining significant levels of *qE*. However, the reduced levels of ATP synthase were not explicitly attributed to translational and/or transcriptional phenomena (Kohzuma et al., 2009).

Here, we demonstrate that this well-known response of dynamic *qE* sensitivity is a key component of dehydration stress memory. Moreover, we provide the first evidence that drought-induced *qE* sensitivity modulation involves gene transcriptional changes. Specifically, we observed reduced expression and a delayed memory response of the gene coding for the γ subunit of CF_0_CF_1_ ATP synthase, the inactivation of which has been shown to result in high non-photochemical quenching in *Arabidopsis* (Dal Bosco et al., 2004). Transcriptional changes in the maize genome were coordinated with attenuation of *ETR* and enhancement of *qE*. Therefore, it can be inferred that the relative resistance of ATP synthase increased between R1 and S2, likely due to reductions in the amount of the enzyme, thereby effecting the observed increase in *qE* sensitivity. It is of significant interest to determine whether the gene networks controlling levels of ATP synthase share regulatory effectors with other gene networks involved in the dehydration stress memory response.

Why photoprotective responses showed delayed memory and were not induced immediately in the first stress may be partly due to C4-mediated carbon-concentration (Rao and Dixon, 2016). Since *P*_N_ did not decline dramatically until S2, carboxylation was still occurring in S1, presumably with CO_2_ previously concentrated in the bundle sheath cells. Further accumulation of CO_2_ was presumably minimal, since stomatal conductance was reduced in S1 and R1, and was reduced even further in S2, when photoprotective responses increased. Comparisons with C3 species would be instructive, as their *P*_N_ may be more likely to display a memory response to repeated dehydration stress. Consistent with this prediction is the observation that the most dramatic difference in the nature of the dehydration stress memory genes in maize versus the C3 eudicot, *A. thaliana*, was that photosynthesis-related genes in *Arabidopsis* displayed [−/−] and [−/+] memory responses, whereas the homologous genes in maize belonged mainly to the delayed [−/=] memory category (Ding et al., 2014).

Changes in other chlorophyll fluorescence parameters give insights into the physiological integration of the dehydration stress memory response. The decrease in φ*PSII*, and φ*CO_2_*, between R1 and S2 can be explained by the concomitant increase in *qE* and decrease in *qP*. The delayed memory pattern of these parameters suggests that closure of PSII reaction centers occurred only after a severe dehydration stress and/or after induction of dehydration stress memory. Consistent with our findings, a study of drought memory over the course of a growing season in a C3 grass species also found enhanced photoprotection of doubly stressed plants (Walter et al., 2011). The coordinated decline in *F*_v_′*/F*_m_′, a measure of the efficiency of PSII if all reaction centers were oxidized (Baker et al., 2007), suggests that the maximal capacity of PSII electron transport was also affected by stress memory, possibly due to damage of PSII reaction centers (Genty et al., 1989; Tyystjärvi and Aro, 1996) or migration of light-harvesting antennae from PSII to PSI (Genty et al., 1990). The increases in *ETR*/*A_G_* and φ*PSII/*φ*CO_2_* suggest that by S2, more energy was being dissipated by non-carboxylative mechanisms (Edwards and Baker, 1993; Loriaux et al., 2013).

All of the photosynthesis and fluorescence parameters exhibited a delayed memory response pattern that correlated remarkably well with the transcriptional response patterns of implicated genes. Thus, meta-module A, representing 2757 co-expressed delayed memory response genes, encoded proteins involved in light harvesting, electron transport, non-photochemical quenching, and in overall photosynthesis including enzymes for the CBB cycle. Genes known to be involved in NPQ, ZE (npq2) and VDE (npq1) (Niyogi et al., 1998), were found in both the M1 and the solo modules, both of which correlated with *qE* and φ*qE*. All else being equal, the increased expression of zeaxanthin epoxidase (npq2) and decreased expression of violaxanthin de-epoxidase (npq1) would presumably cause higher violaxanthin levels and lower zeaxanthin levels in subsequent stresses. Although zeaxanthin is necessary for NPQ (Demmig-Adams and Adams, 2000; Adams et al., 2006), it is not sufficient: both the pH gradient across the thylakoid membrane (Müller et al., 2001) and the PSII protein, Psbs (Li et al., 2000), are also necessary (Takizawa et al., 2008). We found no changes in the expression of Psbs. However, the apparent paradox of putatively lower npq1 but higher *qE* in S2, could be explained by a smaller pool of npq1 that is more highly activated by a reduced pH in the thylakoid lumen (Kramer et al., 1999). The relationship between *qE* and zeaxanthin is likely to be more complex under drought. In support, other studies have also found transcript levels of npq1 and npq2 not to correlate well with short-term responses in these pigments (North et al., 2005).

## Conclusion

Current models posit that short-term physiological responses of plants to environmental stress are predominantly mediated by biochemical changes. Our findings, however, demonstrate that changes in gene expression are also an important component of the stress response mechanism and that short-term photosynthetic and photoprotective responses to repeated dehydration stresses are partially controlled at the transcriptional level. The dehydration stress memory response in maize involves multiple physiological mechanisms that act to protect the photosynthetic machinery while maximizing carbon gain under fluctuating water availability. Our study is novel in that we quantified these mechanisms in detail and in coordination with foliar transcriptomic data from multiply stressed/rehydrated seedlings, allowing for new insights into the organization of the transcriptional and physiological responses involved. Moreover, that a gene for chloroplastic ATP synthase displayed downregulated delayed memory, to our knowledge, provides the first evidence that drought-induced modulation of *qE* sensitivity involves a transcriptional component. That both gene expression-independent and dependent mechanisms drive this and other responses is a novel revelation of how the photosynthetic and photoprotective processes occurring in dehydration stress memory responses are regulated.

Establishing whether genes grouped in a particular meta-module share specific transcription factors’ (TFs) binding motifs might provide additional insights into their memory patterns. However, data on the TFs and their binding sights in the maize genome are currently sparse, which limits analyses of their potential enrichment within a module. Extrapolation from data regarding TF binding sites in other species is impractical, as the grass cistrome is highly divergent from that of *A. thaliana*, and species-specific differences in the response of photosynthesis genes may be significant, even between grass species (Kümpers et al., 2017). Although critical for the transcription of regulated genes, the transcriptional behavior of a TF, alone, cannot explain, or predict, their memory patterns. For example, the memory expression pattern of the TF MYC2 under repeated stress did not correlate with the non-memory expression pattern of its target marker gene *RD22* (Liu et al., 2014), and the (non-memory) expression patterns of the TFs, ABFs, does not correlate with the super-induced memory patterns of their directly regulated *RD29B* or *RAB18* genes (Virlouvet et al., 2014). The roles of the TFs in the memory transcription of their directly regulated targets may need to be established for each case.

Why some genes and responses in these networks showed memory versus delayed memory is unknown, but may reflect cost-benefit trade-offs. For example, fewer genes were involved in the memory response of stomatal regulation, compared to substantially more that were involved in delayed memory responses of photosynthetic and photoprotective processes. Avoiding water loss by changing regulation of relatively fewer genes involved in the memory response of stomatal regulation may have lower costs and immediate benefits, compared to altering expression of substantially more genes involved in photosynthesis, which may require more energy and also come at the cost of an altered photosynthetic apparatus and reduced carboxylation capacity.

Our study demonstrates that stress memory contributes to environmental acclimation, considered plastic and reversible, but, depending on the mechanisms regulating changes in gene expression, it could also contribute to heritable variation in stress responses (Hauser et al., 2011). However, the transcription memory of several tested memory genes lasted 5–7 days after removal of the dehydration stress (Ding et al., 2012), suggesting that the dehydration stress memory is short-term and reversible, unlikely to be preserved *trans*-generationally, and functioning as a mechanism for transcriptional responses during recurring bouts of drought. By temporarily altering expression of specific genes and related physiological parameters, it may facilitate an individual plant’s acclimation and survival under repeated seasonal drought spells. Further studies will provide more understanding of how gene regulatory and physiological networks interact to produce phenotypically integrated drought memory responses in plants, which is fundamental to developing agricultural crops tuned to the rapid environmental changes occurring across the globe.

## Availability of Data

The physiological data used in this study are available from the corresponding author on reasonable request. The raw transcriptome sequence files for watered, S1, and S3 have been uploaded, together with gene expression result files, to NCBI’s Gene Expression Omnibus under sequence number GSE48507.

## Author Contributions

ZA, MF, and SR designed the study and supervised data collection. LV and NL collected the physiological data. SR, TA, and LV analyzed and interpreted the physiological data. ZA and LV conducted the GO-term analyses. CZ and QD conducted the network analyses. LV, ZA, SR, TA, and CZ wrote the manuscript.

## Conflict of Interest Statement

The authors declare that the research was conducted in the absence of any commercial or financial relationships that could be construed as a potential conflict of interest.

## Footnotes

1 https://maizegdb.org/

## Supplementary Material

APPENDIX S1 Supplementary materials and methods.

TABLE S1 List of genes in each module and their degrees in the co-expression network.

TABLE S2 Gene ontology term enrichment analysis for the genes from each meta-module and module of the co-expression network analysis.

TABLE S3 Classification of memory genes into four broad functional categories related to photosynthesis, pigments, stomatal regulation, and production of abscisic acid.
