# Supplementary material for: Dehydration Stress Memory: Gene Networks Linked to Physiological Responses During Repeated Stresses of Zea mays
Source: Front Plant Sci. 2018 Jul 24;9:1058. doi: 10.3389/fpls.2018.01058 (PMC6066539; doi:10.3389/fpls.2018.01058)
Supplement: APPENDIX S1 — Supplementary materials and methods. [file Presentation_1.pdf]

Appendix S1: Supplementary Materials and Methods

Plant growth and treatments

Zea mays L. (cultivar B73) seedlings were grown in soil (Sunshine MVP mixture with addition of one Micromax teaspoon per bushel of sunshine MVP; Sun Gro Horticulture, Canada Ltd.) in the greenhouse with regular watering. Seedlings were removed gently from pots to avoid root damage, and after washing soil away their roots were placed in water, and left to acclimate overnight in covered trays (W, well-watered stage). Leaf RWC was calculated using the formula RWC (%) = ((FW-DW) / (TW-DW)) x 100 where FW, TW and DW correspond, respectively, to the fresh, turgid and dry weights of a piece of leaf tissue (Smart, 1974).

Abscisic acid content

The abscisic acid (ABA) assay was performed as in Liu et al. [1]. Specifically, leaf discs (*ca.* 200 mg fresh weight) were punched from the fourth leaf of four seedlings at W, S1, R1, and S2 stages. The leaf discs were immediately ground in liquid nitrogen and homogenized in 90% (v/v) methanol containing 200 mg L-1 of diethydithiocarbamic acid sodium salt. The extracts were then incubated overnight in a covered, silanized in borosilicate tube in darkness at 4 °C, followed by low-speed centrifugation. The methanolic supernatant was recovered and evaporated, and the residue was dissolved by methanolic Tris buffer (10% methanol, 50 mM Tris, pH 8.0, 1 mM MgCl2 and 150 mM NaCl). An ELISA kit (Agdia, USA) was used for the determination of ABA (pmol g-1 DW) following the manufacturer’s instructions.

Gas exchange and chlorophyll fluorescence

A LI-6400XT infrared gas analyzer, integrated with a 6400-40 fluorescence chamber comprised of a uniform LED light source and a pulse-amplitude-modulation (PAM) chlorophyll *a* fluorometer (LI-COR Inc., Lincoln, NE, USA), was used to collect gas exchange and fluorescence parameters that are related important aspects of photosynthesis. The red modulation LEDs, used for measuring fluorescence parameters, and the red actinic LEDs, used to drive photosynthesis and provide saturation flashes (below), of the 6400-40 are spectrally identical and exhibit peak emission at 632 nm with a full-width-half-maximum of ~20 nm (LI-6400XT Manual, Book 5). While these LEDs excite the *blue edge* of chlorophylls *a* and *b* Q_y transitions

[2], chlorophyll *b* → *a* energy transfer occurs on the 100-200 fs and 10's of ps timescale [3, 4]. Thus, chlorophyll *a* fluorescence (*i.e.* a ns timescale phenomenon) is selectively elicited by the modulation LEDs of the 6400-40 fluorometer. Chlorophyll *a* fluorescence emission exhibits peak wavelengths detectable at 690 nm and 730 nm [5]. However, in leaves, due to significant re-absorbance of the shorter (*i.e.* < 715 nm) wavelengths, fluorescence emission at 690 is weak [6]. The 6400-40 fluorometric detector configuration consists of optical filters that result in preferential detection of fluorescence predominantly at wavelengths longer than 715 nm. It has been demonstrated, however, that 30% and 50% of the minimum fluorescence yield (Φ_F), or *Fo* (below), detected at 730 nm in C3 and C4 species, respectively, is not quenched by non-photochemical quenching (NPQ) [7] in the photosystem (PS) II antenna [5]. It is thus thought that this non-quenched fluorescence emanates from pigment matrices not associated with PSII and very likely represents fluorescence emanating from PSI [5], requiring a corrective protocol (see below).

According to a "lake" model of PSII photophysics [8], PAM chlorophyll *a* fluorescence parameters can be described by fluorescence *yield* (Φ_F) equations that represent ratios of rate constants for processes that compete with one another to quench singlet excited chlorophyll (^{1}Chl*) to the groundstate. The minimum Φ_F obtained after prolonged (*i.e.* hours, overnight, *etc.*) dark-adaptation (*Fo*) can be described as in [8]:

$$Fo = \Phi_F = \frac{\mathrm{kF}}{\Sigma(\mathrm{kF+kd+kISC+kpi}*[\mathrm{qL}=1])} \qquad (1)$$

The parallel, first order rate constants kF, kd, kISC, and kpi correspond to the processes of fluorescence, intrinsic heat dissipation, intersystem crossing from ^{1}Chl* to the triplet state (^{3}Chl*), and intrinsic electron transfer within the PSII reaction center, respectively. The parameter *qL* (see below) represents the proportion of the oxidized, first quinone acceptor (Q_A) [9, 10] in the PSII reaction center ($Q_A/\Sigma(Q_A + Q_A^-)$), and its value is assumed to be 1 after prolonged dark-adaptation. The rate constant for *NPQ*, kNPQ, is not included in Eqn. 1 since prolonged dark-adaptation is assumed to allow this composite of processes [7], including the rapidly reversible component referred to as energy-dependent quenching (qE), state transitions (qT), and quenching due to inhibition (*qI*), to relax ($kNPQ = \Sigma(kqE + kqT + kqI)$). The maximum Φ_F from such a dark-adapted state (*Fm*) is measured by applying a brief (*i.e.* 500-1000

ms) saturating flash of light that is several orders of magnitude higher than full sunlight and can be expressed as:

$$Fm = \Phi_F = \frac{\mathrm{kF}}{\Sigma(\mathrm{kF+kd+kISC})} \qquad (2)$$

The assumption is that the saturating flash causes *qL* to approach zero, such that (kpi**qL*) drops out of the denominator in Eqn. 2. The minimum Φ_F under steady-state illumination (*F'*) is expressed as:

$$F' = \Phi_F = \frac{\mathrm{kF}}{\Sigma(\mathrm{kF+kd+kISC+kpi}*[0\leq \mathrm{qL} \leq]+\mathrm{kNPQ})} \qquad (3)$$

In Eqn. 3 *qL* is assumed to be between 0 and 1 depending upon variability in the intensity of steady-state illumination, *etc*., during which *NPQ* processes are also variably engaged. Thus, in contrast to the expression for *Fo* (Eqn. 1), k*NPQ* is included in the denominator of Eqn. 3. The expression for the maximum Φ_F under steady-state illumination (*Fm'*) is:

$$Fm' = \Phi_F = \frac{\mathrm{kF}}{\Sigma(\mathrm{kF+kd+kISC+kNPQ})} \qquad (4)$$

Fm' is explicitly assumed herein to represent the *true* value of maximum Φ_F under steady-state illumination, as opposed to the value of *apparent Fm'* ($^{A}Fm'$) (see below). The minimum Φ_F during steady-state illumination assuming *qL* = 1 (*Fo'*) is expressed as:

$$Fo' = \Phi_F = \frac{\mathrm{kF}}{\Sigma(\mathrm{kF+kd+kISC+kpi}*[\mathrm{qL}=1]+\mathrm{kNPQ})} \qquad (5)$$

Fo' is obtained by turning off the steady-state actinic light and simultaneously applying a brief pulse (*i.e.* ~7 sec.) of far-red light [6], which preferentially excites PSI, *e.g.* in order to completely oxidize Q_A^- (*i.e.* $qL \rightarrow 1$.). The major component of *NPQ* is *qE* and it quickly (*i.e.* seconds-to-minutes) relaxes in the dark [11]. Ten minutes following a light-to-dark transition,

application of a saturating flash, *e.g.* in order to cause $qL \rightarrow 0$, results in an estimate the maximum Φ_F assuming qE has relaxed (Fm'') [12, 13]:

$$Fm'' = \Phi_F = \frac{\mathrm{kF}}{\Sigma(\mathrm{kF}+\mathrm{kd}+\mathrm{kISC}+\Sigma(\mathrm{kqT}+\mathrm{kqI}))} \qquad (6)$$

Accuracy of the physiologic phenomena reported herein that are based on measurements of the abovementioned fluorescence parameters presupposes accurate determination of Fm', which has been shown rather to be prone to underestimation by traditional “rectangular” flashes many orders of magnitude higher than full sunlight [14-16]. In contrast, an approach based on a multi-phase flash (MPF) of irradiance has been shown to provide valid approximations of Fm' [14]. An MPF involves measuring changes in PAM chlorophyll *a* fluorescence during three contiguous “phases” of change in flash irradiance. Phase 1 involves an increase in irradiance from the steady-state, actinic level to a maximum irradiance for 300 ms, during which a value of $^{A}Fm'$ is obtained. While the Phase 1 maximum irradiance is linearly attenuated for 300 ms during Phase 2, the Φ_F decreases hyperbolically. Nonetheless, the Phase 2 changes in Φ_F are well approximated by a *linear* function when plotted versus the *reciprocal* of the Phase 2 changes in irradiance (Phase 2^{-1}). The Phase 2 changes in Φ_F are an inverse function of Phase 2^{-1}, that is, as Phase 2^{-1} decreases, Phase 2 Φ_F increases. As such, linear regression of the changes in Phase 2 Φ_F and extrapolation to the *y*-intercept provides an estimate of Φ_F at infinite irradiance, a parameter termed *extrapolated* Fm' ($^{E}Fm'$), that is higher than $^{A}Fm'$. It was demonstrated that values of $^{E}Fm'$ more closely approximate Fm' in *Zea mays* and *Helianthus annuus* [14]. For example, it was shown experimentally in *H. annuus,* as well as computationally, that values of $^{A}Fm'$ increase hyperbolically towards an asymptote as a function of increasing Phase 1 irradiance, whereas the corresponding estimates of $^{E}Fm'$ were shown to be not only invariably higher than the corresponding values of $^{A}Fm'$, but also constant over a wide range of Phase 1 irradiances. The results suggested that measurements of $^{A}Fm'$, even at flash irradiances approaching 13,000 µmol m^{-2} s^{-1}, underestimate Fm', a value that could nonetheless be well approximated by estimates of $^{E}Fm'$ over a range of Phase 1 irradiances.

Herein, preliminary MPF experiments on seedlings exposed to 500 µmol m^{-2} s^{-1} of actinic light revealed an important difference in comparison to the previous study with *H. annuus*: the values of $^{A}Fm'$ and $^{E}Fm'$ merged at a Phase 1 irradiance of ~4,000 µmol m^{-2} s^{-1}. These results

indicated that there was no need to estimate *Fm'* using the MPF approach, but that it could be obtained using traditional rectangular flashes.

Combined gas exchange and PAM chlorophyll *a* fluorescence measurements were performed by placing an individual, fully expanded leaf across the 6 cm^2 chamber. The leaf area inside the chamber was recorded prior to gas exchange measurements if the entire chamber area was not covered. The reference CO_2 concentration and leaf temperature were maintained at 380 ppm and 22°C, respectively. The leaf fan speed was set at fast, flow rate was 300 µmol s^{-1}, and gas exchange measurements were done at PAR of 500 µmol photons m^{-2} s^{-1} inside the chamber. Relative humidity of the ambient air was 35-65 %, and no humidity control was used. Seedlings were dark-adapted overnight prior to measurements in hydration states W and R1, but not S1 and S2 due to constraints of the experimental design, since plants were only exposed to dehydration stress in the light [17]. Leaves were gently clamped into the chamber and estimates of *Fo* and *Fm* were measured just prior to, and during the maximum intensity of, a rectangular flash (~4700 µmol photons m^{-2} s^{-1}), respectively. The leaf was then exposed to actinic light of 500 µmol photons m^{-2} s^{-1} for 40 minutes and steady-state gas exchange rates were measured, as were *F'* and *Fm'* by measuring Φ_F just prior to, and during the maximum intensity of, a rectangular flash (~4700 µmol photons m^{-2} s^{-1}). *Fo'* was subsequently measured post-flash and briefly (several seconds) following cessation of actinic illumination while simultaneously illuminating the leaf with far-red light (Baker 2008). Ten minutes following cessation of actinic illumination, *e.g.* in order to allow *qE* to relax, a saturation flash was initiated to estimate *Fm''* (Avenson et al. 2004). Even though seedlings in S1 and S2 hydration states could not be dark-adapted, precluding estimation of *Fo*, *Fm,* as well as their derivative parameters (*i.e. NPQ, Fv/Fm*), *qE* is the major component of *NPQ* [7] and does not require estimation of *Fm* (see below). Thus, *qE* was estimated during all four hydration states.

Physiologic phenomena that are based on measurements of the abovementioned fluorescence parameters were calculated as described in Baker et al. [18], using the nomenclature in Table 1. Briefly, the maximum quantum efficiency of PSII photochemistry (*Fv/Fm*) was calculated as:

$$Fv/Fm = (Fm - Fo)/Fm \qquad (7)$$

Fv/Fm is a robust parameter that has been previously shown to be conserved across a diverse range of 43 species (mean *Fv/Fm* = 0.83 ± 0.022) [19], and it is a sensitive indicator or stress [20]. As mentioned above, 30% and 50% of *Fo* detected at 730 nm in C3 and C4 species, respectively, is thought to emanate from PSI. However, while both photosystem PSI and PSII emit fluorescence at these detected wavelengths, the physiologic phenomena based on PAM chlorophyll *a* fluorescence parameters are predicated on measuring fluorescence emission *solely* from PSII [5, 21]. A previous study demonstrated prior to, and post, correction for contributions of PSI fluorescence, that estimates of *Fv/Fm* in *Z. mays* were 0.76 and 0.87, respectively [6], thus demonstrating that correction of fluorescence parameters for contributions from PSI fluorescence is essential for accurate determination of PAM chlorophyll *a* fluorescence parameters (Eqns. 1 – 6). Herein, fluorescence parameters were corrected for contributions from PSI fluorescence by assuming that 50% of the measured *Fo'* values represented fluorescence emanating from PSI (Note: it was not possible to measure, and thereby correct for, PSI fluorescence as previously described [6] using seedlings during S1 and S2 because the correction approach requires estimates of *Fm* and *Fo*; thus corrections based on *Fo'*, a parameter measured during all hydration states, were performed). The difference between the *measured Fo'* (*i.e*. 50% contributions from both PSII and PSI) and the *corrected Fo'* (*i.e*. 50% of the measured *Fo'* and assumed to represent fluorescence emanating solely from PSII) was assumed to represent PSI fluorescence. The estimated PSI fluorescence was subsequently subtracted from measured values of *Fm*, *Fo*, *F'*, *Fm'*, and *Fm"* in order to correct these parameters. Using this approach for dark-adapted plants of W and R1 hydration states, pre- and post-corrected mean values of *Fv/Fm* were 0.74 and 0.83, respectively, very similar to observations in *Z. mays* using an alternative approach for correction [6].

The operating efficiency of *PSII* (*φPSII*) is measured under illuminated conditions, it can be used to estimate a wide assortment of important photosynthetic phenomena, and it is calculated as:

$$\varphi PSII = (Fm' - F')/Fm' = \frac{\mathrm{kpi}*[0\leq \mathrm{qL}\leq 1]}{\Sigma(\mathrm{kF}+\mathrm{kd}+\mathrm{kISC}+\mathrm{kpi}*[0\leq \mathrm{qL}\leq 1]+\mathrm{kNPQ})} \quad (8)$$

It is evident from inserting Φ_F's described by Eqns. 3 and 4 into Eqn. 8, and performing the necessary algebraic manipulations (right side of the expression), that *φPSII* can be impacted, in

part, by the ability of PSII reaction centers to transfer electrons to secondary electron acceptors (*i.e*. $Q_A^- \rightarrow Q_B$) [9, 10], a direct reflection of the value of *qL*, which varies between 0 and 1 in Eqn.8. *qL* can be derived from Φ_F parameters according to Kramer et al. [8]:

$$qL = qP * (Fo'/F') \qquad (9)$$

Note that *qP* is calculated as in Genty et al. [22]:

$$qP = (Fm' - F')/(Fm' - Fo') \qquad (10).$$

qP has historically been reported to be representative of the proportion of open PSII reaction centers, but it should be stressed that *qP* represents the proportion of open centers *only* in the context of a “puddle” model of PSII photo-physics, whereas it has been shown that PSII photo-physics are best described rather by a lake model [8, 9]. Nonetheless, herein we chose to report *qP* since it behaved qualitatively similar to *qL* (data not shown). While *φPSII* can also be impacted by *NPQ* processes, it is not explicitly evident from Eqn. 8 the extent to which *NPQ* does so. In contrast, the PSII maximum efficiency under illuminated conditions (*Fv'/Fm'*), a parameter, unlike *φPSII*, that is based on the assumption that *qL* = 1, provides explicit information about how *φPSII* is impacted by changes in *NPQ* and is calculated as [18]:

$$Fv'/Fm' = (Fm' - Fo')/Fm' = \frac{\mathrm{kpi}*[\mathrm{qL}=1]}{\Sigma(\mathrm{kF}+\mathrm{kd}+\mathrm{kISC}+\mathrm{kpi}*[\mathrm{qL}=1]+\mathrm{kNPQ})} \qquad (11)$$

φPSII can be used to quantify electron transfer (ETR), a process involving oxidation of water at PSII and, via a linked series of redox reactions, reduction of $NADP^+$ at the reducing side of PSI:

$$ETR = \varphi PSII \times PAR \times 0.5 \times \alpha_{leaf} \qquad (12)$$

We calculated *ETR* [23] assuming equal distribution of light energy between PSI and PSII (0.5) and a leaf absorbance (α_{leaf}) of 0.84 (Earl and Tollenaar 1998).

Comparison of ETR with certain gas exchange parameters, as well as with other fluorescence-derived parameters, can provide unique information about nuanced aspects of photosynthesis. Based on different assumptions of the variable oxidation state of PSII reaction centers, we estimated two expressions representative of the rapidly reversible component of *NPQ*, energy-dependent quenching (*qE*) [7, 12] and the *quantum yield* of energy dependent quenching (φqE) [24]:

$$qE = (Fm'' - Fm')/Fm'' \qquad (13)$$

and

$$\varphi qE = ((Fm'' - Fm')/Fm'') \times (Fs / Fm') \qquad (14)$$

The former is predicated on $qL = 0$, whereas the latter expression accommodates the intrinsic variability in *qL* (*i.e*. between 0 and 1) that occurs under fluctuating light, *etc*. The ratio of *qE* to *ETR* (*qE/ETR*) was calculated in order to detect the possibility of a previously reported stress-induced phenomenon termed *qE* sensitivity [8, 13]. It was previously shown that biochemically perturbing CO_2 metabolism (*i.e*. by artificially lowering CO_2 availability to the leaf in order to mimic drought stress) significantly reduced ETR, and yet higher levels of *qE* were nonetheless generated, *e.g. qE* became more sensitive to ETR upon biochemical perturbation [8, 13]. The ratio of ETR to gross CO_2 assimilation (P_G) provides an estimate of the electron requirement of the carboxylative reactions [14, 25], a parameter that can be indicative of alternative energy-consumptive reactions (*i.e*. photorespiration). P_G corresponds to the total amount of CO_2 that is assimilated and was calculated as:

$$P_G = P_N + R \qquad (15)$$

P_N represents net photosynthesis and *R* corresponds to dark respiration, which was obtained by measurement of P_N in the dark for the dark-adapted plants. P_G was also used to estimate the quantum yield of CO_2 assimilation (φCO_2) [25]:

$$\varphi CO2 = P_G / (PAR \times \alpha_{leaf}) \qquad (16)$$

The ratio of $\varphi PSII:\varphi CO_2$ quantitatively assesses the coupling between ETR and CO_2 assimilation, which can be indicative of changes in: 1) α_{leaf}; 2) alternative sinks for energy other than CO_2 assimilation; and 3) the partitioning of absorbed light to PSII [25].

Literature Cited

1. Liu N, Ding Y, Fromm M, Avramova Z, . Endogenous ABA extraction and measurement from *Arabidopsis* eaves. Bio-protocol 4(19): e1257. 2014.
2. Lichtenthaler HK, Buschmann C. Chlorophylls and carotenoids: measurement and characterization by UV-VIS spectroscopy. In *Current Protocols in Food Analytical Chemistry*. John Wiley & Sons, Inc.; 2001
3. Avenson TJ, Ahn TK, Zigmantas D, Niyogi KK, Li Z, Ballottari M, Bassi R, Fleming GR. Zeaxanthin radical cation formation in minor light-harvesting complexes of higher plant antenna. J Biol Chem. 2008; 283**:**3550-3558.
4. Croce R, Müller MG, Bassi R, Holzwarth AR. Chlorophyll b to chlorophyll a energy transfer kinetics in the CP29 antenna complex: A comparative femtosecond absorption study between native and reconstituted proteins. Biophys J. 2003; 84**:**2508-2516.
5. Genty B, Wonders J, Baker NR. Non-photochemical quenching of Fo in leaves is emission wavelength dependent: consequences for quenching analysis and its interpretation. Photosynthesis Res. 1990; 26**:**133-139.
6. Pfündel EE, Klughammer C, Meister A, Cerovic ZG. Deriving fluorometer-specific values of relative PSI fluorescence intensity from quenching of F0 fluorescence in leaves of *Arabidopsis thaliana* and *Zea mays*. Photosynthesis Res. 2013; 114**:**189-206.
7. Müller P, Li X-P, Niyogi KK. Non-photochemical quenching. A response to excess light energy. Plant Physiol. 2001; 125**:**1558.
8. Kramer DM, Johnson G, Kiirats O, Edwards GE. New fluorescence parameters for the determination of QA redox state and excitation energy fluxes. Photosynthesis Res. 2004; 79**:**209.
9. Lavergne J, Trissl HW. Theory of fluorescence induction in photosystem II: derivation of analytical expressions in a model including exciton-radical-pair equilibrium and restricted energy transfer between photosynthetic units. Biophys J. 1995; 68**:**2474-2492.

10. Schatz GH, Brock H, Holzwarth AR. Kinetic and energetic model for the primary processes in photosystem II. Biophys J. 1988; 54**:**397-405.

11. Crofts AR, Yerkes CT. A molecular mechanism for *qE*-quenching. FEBS Letters. 1994; 352**:**265-270.

12. Avenson TJ, Cruz JA, Kramer DM. Modulation of energy-dependent quenching of excitons in antennae of higher plants. Proc Natl Acad Sci USA. 2004; 101**:**5530-5535.

13. Kanazawa A, Kramer DM. In vivo modulation of nonphotochemical exciton quenching (NPQ) by regulation of the chloroplast ATP synthase. Proc Natl Acad Sci USA. 2002; 99**:**12789-12794.

14. Loriaux SD, Avenson TJ, Welles JM, McDermitt DK, Eckles RD, Riensche B, Genty B. Closing in on maximum yield of chlorophyll fluorescence using a single multiphase flash of sub-saturating intensity. Plant, Cell Environ. 2013; 36**:**1755-1770.

15. Markgraf T, Berry J. Measurement of photochemical and nonphotochemical quenching: correction for turnover of PS2 during steadystate photosynthesis. In *Current Research in Photosynthesis.* Edited by Baltscheffsky M. Dordrecht, the Netherlands.: Kluwer Academic Publishers; 1990: 279–282

16. Earl HJ, Ennahli S. Estimating photosynthetic electron transport via chlorophyll fluorometry without Photosystem II light saturation. Photosynthesis Res. 2004; 82**:**177-186.

17. Ding Y, Virlouvet L, Liu N, Riethoven J-J, Fromm M, Avramova Z. Dehydration stress memory genes of *Zea mays*; comparison with *Arabidopsis thaliana*. BMC Plant Biol. 2014; 14**:**141.

18. Baker NR, Harbinson J, Kramer DM. Determining the limitations and regulation of photosynthetic energy transduction in leaves. Plant, Cell Environ. 2007; 30**:**1107-1125.

19. Bjorkman O, Demmig B. Photon yield of O2 evolution and chlorophyll fluorescence characteristics at 77 K among vascular plants of diverse origins. Planta. 1987; 170**:**489-504.

20. Tyystjärvi E, Aro EM. The rate constant of photoinhibition, measured in lincomycin-treated leaves, is directly proportional to light intensity. Proc Natl Acad Sci USA. 1996; 93**:**2213-2218.

21. Schreiber U, Hormann H, Neubauer C, Klughammer C. Assessment of photosystem II photochemical quantum yield by chlorophyll fluorescence quenching analysis. Funct Plant Biol. 1995; 22:209-220.

22. Genty B, Briantais J-M, Baker NR. The relationship between the quantum yield of photosynthetic electron transport and quenching of chlorophyll fluorescence. Biochimica et Biophysica Acta. 1989; 990:87-92.

23. Lal A, Edwards G. Analysis of inhibition of photosynthesis under water stress in the C4 species *Amaranthus cruentus* and *Zea mays*: Electron transport, CO_2 fixation and carboxylation capacity. Funct Plant Biol. 1996; 23:403-412.

24. Ahn TK, Avenson TJ, Peers G, Li Z, Dall'Osto L, Bassi R, Niyogi KK, Fleming GR. Investigating energy partitioning during photosynthesis using an expanded quantum yield convention. Chemical Physics. 2009; 357:151-158.

25. Edwards GE, Baker NR. Can CO2 assimilation in maize leaves be predicted accurately from chlorophyll fluorescence analysis? Photosynthesis Res. 1993; 37:89-102.
